# Micropower Impulse Radar: A Novel Technology for Rapid, Real-Time Detection of Pneumothorax 

**DOI:** 10.1155/2011/279508

**Published:** 2011-05-30

**Authors:** Phillip D. Levy, Tracey Wielinski, Alan Greszler

**Affiliations:** ^1^Medical Center Emergency Services, 4201 St. Antoine, Detroit, MI 48201, USA; ^2^PneumoSonics Inc., 1771 E. 30th Street, Cleveland, OH 44114, USA

## Abstract

Pneumothorax detection in emergency situations must be rapid and at the point of care. Current standards for detection of a pneumothorax are supine chest X-rays, ultrasound, and CT scans. Unfortunately these tools and the personnel necessary for their facile utilization may not be readily available in acute circumstances, particularly those which occur in the pre-hospital setting. The decision to treat therefore, is often made without adequate information. In this report, we describe a novel hand-held device that utilizes Micropower Impulse Radar to reliably detect the presence of a pneumothorax. The technology employs ultra wide band pulses over a frequency range of 500 MHz to 6 GHz and a proprietary algorithm analyzes return echoes to determine if a pneumothorax is present with no user interpretation required. The device has been evaluated in both trauma and surgical environments with sensitivity of 93% and specificity of 85%. It is has the CE Mark and is available for sale in Europe. Post market studies are planned starting in May of 2011. Clinical studies to support the FDA submission will be completed in the first quarter of 2012.

## 1. Introduction

The ability to rapidly identify a pneumothorax (PTX) at the point of care for trauma victims can be life saving. This is particularly true in the military setting where undetected tension PTX is thought to contribute to death in up to 4% of fatal combat cases [1]. Accurate diagnosis of PTX in the prehospital setting depends on physical examination skills which include the ability to look for respiratory distress, jugular venous distension, or tracheal deviation, listen for diminished lung sounds, and feel for crepitus or broken ribs. Detection of such findings however can be challenging [2–4] even when physician providers are involved in patient assessment [5]. Consequently, prehospital protocols often incorporate a low-threshold for intervention when a PTX is suspected clinically [6–10]. 

Needle decompression, the procedure most commonly performed prehospital for emergent treatment of PTX, is not benign and has the potential to induce substantial morbidity when applied inappropriately [6, 7]. The existence of a quick, practical, easy to use method of diagnosing PTX would greatly improve the margin of error for prehospital providers and facilitate the use of precise, directed intervention for individuals with thoracoabdominal injury. Portable lung ultrasound (US) has high sensitivity and specificity for detection of PTX [11–14] and has been proposed as modality capable of fulfilling this need [15–18]. Performance of lung US, however, requires advanced training and its accuracy is highly operator dependent making it suboptimal for use by basic field medics [19]. Moreover, there are issues with existing portable US equipment including cost, weight, and durability which preclude broad adoption by regionally funded emergency medical service (EMS) providers. 

**Figure 1:**
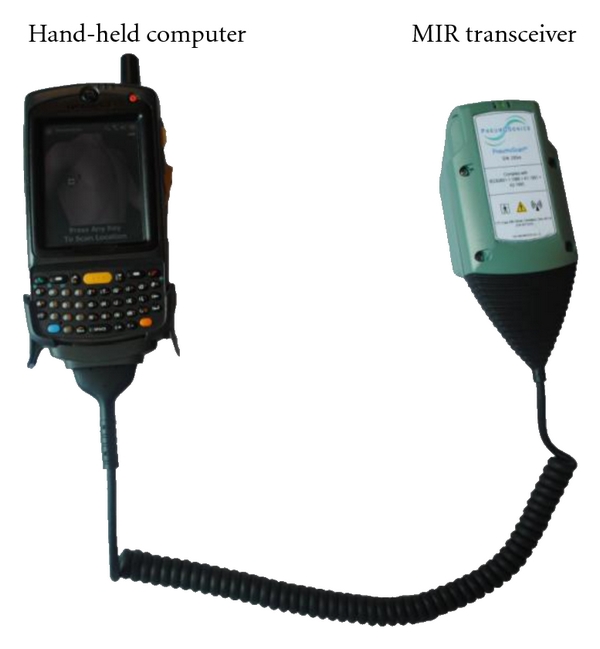
PneumoScan device.

**Figure 2:**
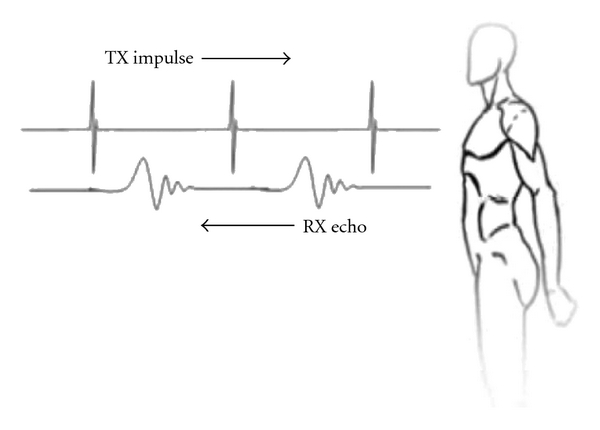
Micropower impulse radar signaling.

A novel alternative to PTX detection has been developed by PneumoSonics Inc. (Cleveland, OH, USA). Based on a technology called micropower impulse radar (MIR), the “PneumoScan” (Figure 1) is a portable device that emits ultrashort radar pulses (<1 ns) with pulse repetition rates on the order of 4 MHz. The device utilizes the same ultrashort pulse circuitry for time gating, with a 33 gigasample-per-second transient digitizer that allows the detection of reflective surfaces in air with spatial accuracy of approximately 5 mm (Figure 2). The radar return signals are digitized and immediately stored for real-time analysis using a proprietary algorithm. 

## 2. Micropower Impulse Radar Technology

Micropower impulse radar technology has been licensed by PneumoSonics from Lawrence Livermore National Laboratories for use in medical devices. The technology uses very short ultrawideband (UWB) pulses that penetrate the body cavity. Returned echoes are a result of the type of medium that is encountered along the path of the pulse. The magnitude of the reflection and transmission coefficients depends on the relative permittivity of the structures. Using the fact that air has a different permittivity than normal body habitus, it is possible to use MIR for the detection of a pneumothorax. Also, as long as the tissues involved (and their permittivity) are known, the distances between reflections can be calculated by measuring the time and performing simple mathematics. The advantages of producing and detecting very brief radar impulses are considerable.

- The target echoes return a tremendous amount of information. With short pulses, the system operates across a wide band of frequencies, giving high resolution and accuracy. The system is also less susceptible to interference from other radars.
- Battery current is drawn only during the short time the system is pulsed, so power requirements are extremely low (<0.1 Watt).
- The microwave power associated with pulsed transmission is exceedingly low (averaging tens of microwatts) and is medically safe.

The advantages of using UWB itself are significant in that it is possible to penetrate differing tissue densities. By optimizing the center frequency, UWB signals are able to distinguish tissue types (e.g., fat, muscle, bone, pooled blood) from air and each other. Additionally, the system readily penetrates clothing allowing the device to be used in the field or in the hospital setting quickly without having to disrupt the patient.

## 3. Using the PneumoScan

**Figure 3:**
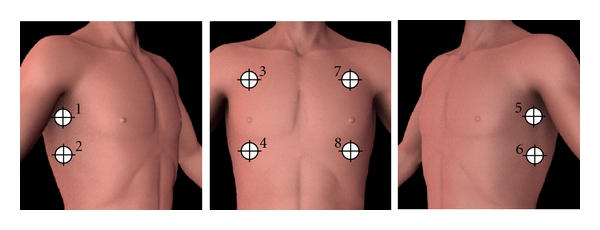
Pneumoscan data acquisition points.

**Figure 4:**
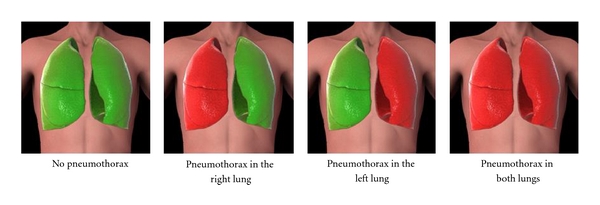
Example of real-time Pneumoscan data interpretation and report.

Use of the PneumoScan is straightforward and total scan time is less than 1 minute. The device is operated by acquiring signals from a transceiver placed at eight pre-specified sites along the anterior thorax (Figure 3). These locations are designed to allow rapid scanning of the entire chest cavity to isolate the PTX to a specific side of the body. Data are sent in real time to be analyzed by a program housed in the connected, hand-held computer. Skin contact is unnecessary as the MIR pulses can penetrate through clothing. The device emits an audible and visual signal when a scan is complete. Once all scans are completed, the PneumoScan analyzes the echoes and immediately displays results to the user (Figure 4) indicating the presence and location (side) of a PTX.

## 4. Simulated Pneumothorax

**Figure 5:**
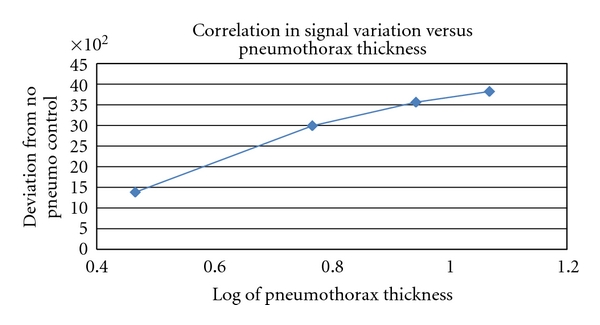
Correlation of scan analysis as a function of simulated pneumothorax thickness.

**Figure 6:**
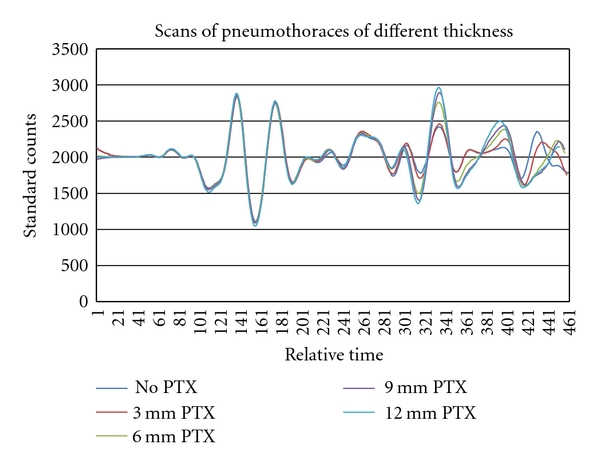
Scan results of various thicknesses simulated pneumothoraces.

Initial study of MIR technology for PTX detection was conducted using a phantom system (concentric discs filled with water or air) developed specifically to simulate pneumothoraces of varying thickness. As shown in Figure 5, MIR signals change as a function of air pocket thickness (tested range: 3 to 12 mm). The root mean square deviation of the response signal obtained in phantoms with and without simulated PTX was calculated. A log normal relationship between the thickness of the PTX and the deviation from the control phantom was noted. These MIR transmission/reflection characteristics of the system can be applied to a one-dimensional projection of PTX volume. Each scan was then compared against the phantom system with no PTX present. The differences (Figure 6) show up at approximately point 281, which corresponds to the starting depth of the pneumothorax in the phantom based on time of flight calculations. Using proper filters and analysis methods, we can ascertain the depth of the PTX, how large the pneumothorax is based on the time of travel to the next dielectric layer, and detect edges to allow reconstruction of the pneumothorax shape and size.

## 5. Preliminary Clinical Data

Initial clinical testing was performed on patients who presented to either of two Level 1 Trauma centers (Detroit Receiving Hospital and Sinai-Grace Hospital, both located in Detroit, MI, USA) with a blunt or penetrating chest injury. Using a prototype of the PneumoScan, a reading was taken prior to any intervention by the Emergency Department staff and confirmed with a chest X-ray (CXR) or computed tomography (CT) scan. We then evaluated the device's diagnostic capabilities based on the following definitions.

- True positive: MIR device identifies presence of a clinically significant PTX and the correct lung, as verified by CXR or CT.
- True negative: Both MIR and CXR or CT determine no clinically significant PTX.
- False positive: MIR identifies presence of a clinically significant PTX, while CXR or CT does not.
- False negative: CXR or CT identifies a clinically significant PTX but MIR does not or MIR identifies the incorrect lung when a PTX is present while not detecting the proper lung.

Reasonable sensitivity (93%) and specificity were found (Table 1) prompting further device refinement and a follow-up study in patients scheduled for elective cardiothoracic surgery, who, by nature of their procedure, were at risk for development of a PTX. Using the present, commercially developed version of the PneumoScan, a reading was taken at all 8 acquisition points during the pre-, intra-, and postoperative period and the presence of a PTX was visually confirmed by the operating surgeon (used, for purposes of this study, as the gold-standard for diagnosis). Data were processed off line with double blinding of clinical findings and device results. Sensitivity was equivalent to the preliminary trauma study (Table 1) but specificity was slightly lower (Table 1). Of note, sensitivity of the PneumoScan in both studies was far better than that which has been reported for CXR (28–75%) [13, 20–23].

## 6. Device Availability

While the PneumoScan has not yet been approved by the Food and Drug Administration (FDA) for use in the United States (USA), it is CE Marked for distribution in Europe. Postmarket release studies are planned for Europe in May of 2011, which will provide important data on real-world performance of the device when used in the trauma setting. Submission to the FDA is targeted for January of 2012, pending completion of a definitive, double-blinded, pre/posttrial of the PneumoScan in patients undergoing lung biopsy procedures. Set to begin at three academic medical centers in the USA, this trial is designed to demonstrate device sensitivity and specificity of 95% with confidence interval of 88.7% to 98.4%; assuming a 3% dropout rate, a total of 345 subjects will be prospectively enrolled.

## 7. Future Directions

With improved antenna design and an advanced algorithm, the PneumoScan may be able to provide information on the specific location and volume of a PTX. Further refinements can also provide within-patient monitoring to allow rapid assessment of changes in a patient's status and more precise quantification of posttreatment PTX resolution. The latter could dramatically reduce the need for repeated CXR thus minimizing compounded exposure to potentially harmful ionizing radiation. 

**Figure 7:**
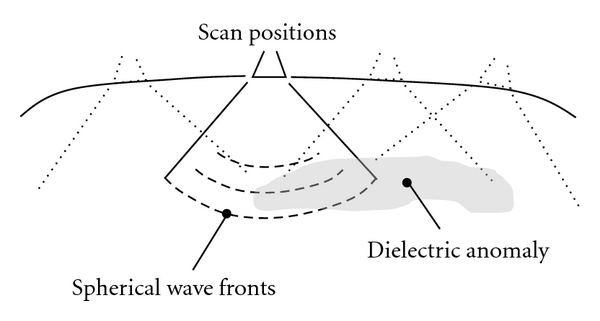
Microimpulse radar signal propagation in the body.

At present, the UWB MIR antenna and electronics provide data that represents a one-dimensional volumetric representation of dielectric gradient into the body. However, the nature of the mechanism that couples the energy into the body with the horn type of antenna currently used in the PneumoScan is such that the “direction” of propagation is essentially hemispheric. That is, a spherical wavefront penetrates the body as shown in Figure 7 centered at the antenna face, and propagates radially, returning a reflection from dielectric differences in the form of an expanding spherical half-sphere and its intersection with those differences. Anomalies in the dielectric as typified by a PTX are traversed radially across their entirety or a portion of their volume, depending upon their size. 

**Figure 8:**
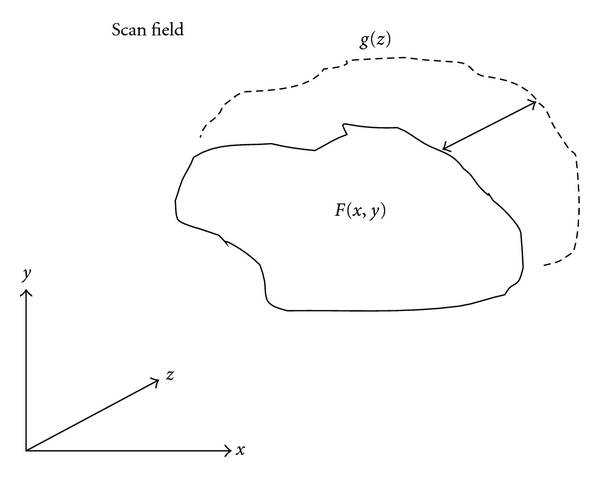
Microimpulse radar scan field and volumetric scatter.

Scans can be taken across the surface of the body and the inversion of their response can provide an accurate depiction of the volume of the anomaly. Inverse scattering can then be used to recover volume and to some extent, the image of any scattering volume within the body, given an appropriate choice of data acquisition and processing techniques. As shown in Figure 8, the form of a dielectric object can be generated by assuming a scattering area *F*(*x*, *y*) that is nearly planar with a gradient in the *z*-direction, *g*(*z*). The processing itself is quite geometric but unlike the Radon or backprojection methods required in CT, it is based on volumetric integration, not full tomographic inversion. 

Additional scan positions provide more detail and can help clarify geometrically complex volumetric interactions. Optimization of volume assessment with PneumoScan, therefore, may require a refinement in how data are acquired, specifically the number and location of scan performance. To better understand this, a pilot study of trauma patients is currently being conducted at Massachusetts General Hospital (Boston, MA, USA) which compares PneumoScan data with PTX volume as quantified from multi detector CT images using a proprietary computer-aided volumetry scheme [24].

## 8. Conclusion

PSI has developed a point-of-care, noninvasive, portable, lightweight, low-power, diagnostic tool for detecting PTX. Based on novel, MIR technology, preliminary data for the PneumoScan are encouraging with a sensitivity of 93% and a specificity of at least 85%. Further study of the PneumoScan is in progress and submission for FDA approval is planned for early 2012. The PneumoScan has been CE Marked and is presently available for clinical use in Europe.

## Figures and Tables

**Table 1:** Preliminary device performance.

	Clinical testing results	
	Trauma study	Surgical study
Total patients (Sides)	53 (106)	50 (100)
Sensitivity	93%	93%
Specificity	89%	84%
Location (left/right) accuracy	93%	100%
